# Development of real-time reverse transcriptase qPCR assays for the detection of Punta Toro virus and Pichinde virus

**DOI:** 10.1186/s12985-016-0509-3

**Published:** 2016-03-31

**Authors:** Christopher P. Stefan, Kitty Chase, Susan Coyne, David A. Kulesh, Timothy D. Minogue, Jeffrey W. Koehler

**Affiliations:** Diagnostic Systems Division, United States Army Medical Research Institute of Infectious Disease, Fort Detrick, MD USA

**Keywords:** Real-time RT-qPCR, Pichinde virus, Punta Toro virus, Bunyaviruses, Arenaviruses

## Abstract

**Background:**

Research with high biocontainment pathogens such as Rift Valley fever virus (RVFV) and Lassa virus (LASV) is expensive, potentially hazardous, and limited to select institutions. Surrogate pathogens such as Punta Toro virus (PTV) for RVFV infection and Pichinde virus (PICV) for LASV infection allow research to be performed under more permissive BSL-2 conditions. Although used as infection models, PTV and PICV have no standard real-time RT-qPCR assays to detect and quantify pathogenesis. PTV is also a human pathogen, making a standardized detection assay essential for biosurveillance. Here, we developed and characterized two real-time RT-qPCR assays for PICV and PTV by optimizing assay conditions and measuring the limit of detection (LOD) and performance in multiple clinical matrices.

**Methods:**

Total nucleic acid from virus-infected Vero E6 cells was used to optimize TaqMan-minor groove binder (MGB) real-time RT-qPCR assays. A 10-fold dilution series of nucleic acid was used to perform analytical experiments with 60 replicates used to confirm assay LODs. Serum and whole blood spiked with 10-fold dilutions of PTV and PICV virus were assessed as matrices in a mock clinical context. The Cq, or cycle at which the fluoresce of each sample first crosses a threshold line, was determined using the second derivative method using Roche LightCycler 480 software version 1.5.1. Digital droplet PCR (ddPCR) was utilized to quantitatively determine RNA target counts/μl for PTV and PICV.

**Results:**

Optimized PTV and PICV assays had LODs of 1000 PFU/ml and 100 PFU/ml, respectively, and this LOD was confirmed in 60/60 (PTV) and 58/60 (PICV) positive replicates. Preliminary mock clinical LODs remained consistent in serum and whole blood for PTV and PICV at 1000 PFU/ml and 100 PFU/ml. An exclusivity panel showed no cross reaction with near neighbors.

**Conclusions:**

PTV and PICV Taq-man MGB based real-time RT-qPCR assays developed here showed relevant sensitivity and reproducibility in samples extracted from a variety of clinical matrices. These assays will be useful as a standard by researchers for future experiments utilizing PTV and PICV as infection models, offering the ability to track infection and viral replication kinetics during research studies.

**Electronic supplementary material:**

The online version of this article (doi:10.1186/s12985-016-0509-3) contains supplementary material, which is available to authorized users.

## Background

Both Rift Valley fever virus (RVFV) and Lassa virus (LASV) are highly pathogenic viruses endemic to Africa. RVFV, within the *Bunyaviridae* family, is a mosquito-borne, biosafety level (BSL)-3 select agent of major public health and economic concern, affecting humans and livestock throughout Africa [1, 8–10, 22] and the Arabian Peninsula [21, 25]. LASV, within the *Arenaviridae* family, is a BSL-4 select agent responsible for approximately 500,000 infections yearly in West Africa [5, 7]. Both of these viruses can result in a hemorrhagic fever syndrome and can cause large outbreaks in endemic regions.

Research with either of these pathogens is hazardous, expensive, and limited to studies at select institutions by approved individuals. As such, BSL-2 infection models for both viruses have been developed: Punta Toro virus (PTV) for RVFV infection and Pichinde virus (PICV) for LASV infection. While these models have been used for multiple pathogenesis and therapeutics studies [3, 6, 11–15, 19, 24, 26], there are no real-time RT-qPCR assays described in the literature for these viruses. The availability of well characterized assays to monitor viral replication kinetics would aid these research efforts.

PTV infection in mice [13] and hamsters [3, 11, 24] results in disease similar to RVFV infection in humans and is an established BSL-2 surrogate infection model for RVFV. PTV, a mosquito-transmitted bunyavirus, typically causes a mild and self-limiting infection in humans but may progress to an acute, febrile illness [4]. Both RVFV and PTV consist of three RNA segments: the small (S), medium (M), and large (L) segments [18, 29]. The L segment encodes the viral polymerase, the S segment contains the nucleoprotein and the nonstructural protein NSs, and the M segment encodes the two glycoproteins Gn and Gc as well as the nonstructural protein NSm.

PICV causes a similar disease in hamsters [6, 12, 14, 26] and guinea pigs [15, 17, 20] as LASV infection in humans. Both PICV and LASV are arenaviruses with a genome comprised of the L and S RNA segments. The L segment encodes the viral polymerase and the Z protein, and the S segment encodes the nucleoprotein and the glycoprotein precursor GPC which is cleaved to yield the glycoproteins GP1 and GP2.

In this study, we designed two TaqMan-based real-time RT-qPCR assays for detection of PTV and PICV. These assays were characterized and evaluated using cell culture supernatant from PTV or PICV infected cells and mock clinical samples. Overall, these assays could benefit the scientific community using animal models as surrogates for RVFV and LASV infection as well as biosurveillance for PTV infections in humans.

## Methods

### Viruses and cells

The Adames strain of PTV and the CO AN 4763 strain of PICV were provided by Dr. Robert Tesh (World Reference Center for Emerging Viruses and Arboviruses, Galveston, TX). Each virus was initially passaged in Vero E6 cells to generate stock virus from the cell culture supernatant. Virus stock titers were determined by standard plaque assay using 0.6 % (*w/v*) SeaKem ME agarose (Lonza, Basel, Switzerland) and a secondary overlay containing 5 % neutral red (ThermoFisher, Waltham, MA). Vero E6 cells were maintained in complete Eagle’s Minimum Essential Medium (cEMEM, Lonza, Basel, Switzerland) supplemented with 10 % (*v/v*) fetal bovine serum (ThermoFisher), 100 U/ml penicillin G (ThermoFisher), and 100 mg/ml streptomycin (ThermoFisher). Cells were incubated at 37 °C with 5 % CO_2_.

### Sample preparation methods

RNA from the cell culture supernatant was extracted using TRIzol LS (ThermoFisher) and the Qiagen EZ1 robot with the EZ1 Virus Mini Kit (Qiagen, Valencia, Ca) according the manufacturer’s directions. Sensitivity testing was conducted for each assay using total nucleic acid isolated from cell culture supernatants from PICV and PTV infected Vero E6 cells. These supernatants were previously titered by plaque assay, so the limit of detection (LOD) was determined based on the number of PFU/ml before sample extraction with the EZ-1 kits. For analytical experiments, extracted RNA was serially diluted 10-fold into water, and the diluted RNA was run with each assay in triplicate. The preliminary LOD was determined based on 3/3 replicates being positive (<40 Cq), and 60 replicates at this preliminary LOD was conducted for LOD confirmation. For mock clinical samples, cell culture supernatant was 10-fold serially diluted in water, serum, and whole blood (BioreclamationIVT, Baltimore, MD), and each dilution was extracted using TRIzol LS and the EZ1 (Qiagen) according to the manufacturer’s instructions.

### Real-time RT-qPCR assay design

Primers and TaqMan-minor groove binder (MGB) probe pairs were designed for PICV and PTV using Primer Express version 2.0 (Applied Biosystems, Foster City, CA) and AlleleID 7.73 (PREMIER Biosoft, Palo Alto, CA). Primer/probe pairs (see Table 1) were designed for PICV (L segment, GenBank# JN378748; S segment, GenBank# JN378747) and PTV (S segment, GenBank# EF201835; M segment, GenBank# DQ363407.1; L segment, GenBank# DQ363408.1). Primers and probe were ordered from ThermoFisher. Initial primer down selection was accomplished by testing for amplicon formation using purified nucleic acid from virus-infected cell culture supernatant and SYBR Green (ThermoFisher), diluted according to manufacturer’s protocol. Resultant amplicons were run on an ethidium bromide gel; primer pairs were selected based on a single, clean PCR product of the correct size.

**Table 1:** Primers for PTV and PICV

Virus	Primers/probe	Sequence (5’-3’)	Conc. (μM)	Amplicon
	F3512	CATGTGTGGCCCCCATTT	0.5	63 bp
PICV	R3574	TCAGTTGTTAGGCAAAGTGGTCTT	0.5	
	P3532S-MGB	6FAM-AATGGTCCATTGACACGG-MGBNFQ	0.2	
	F430	CAGATAGCTGCTGCCATTTTACA	0.5	66 bp
PTV	R495	GCTTTTAAGTTTCCCAGCCAAA	0.5	
	P454S-MGB	6FAM-CTCATTATTGTGGGCTCAT-MGBNFQ	0.2	

For analytical and mock clinical analysis SuperScript One-Step RT-PCR System with Platinum *Taq* DNA polymerase Kit (ThermoFisher) was utilized. 20 μl experiments were performed with 5 μl of extracted RNA described above, 10 μl of 2X SuperScript One-Step RT-PCR master mix, and 0.4 μl of SuperScript II Reverse Transcriptase and Platinum® *Taq* DNA Polymerase mix. The primers and probes used are listed in Table 1. Final concentrations of 0.5 μM primer, 0.2 μM probe, 4 mM MgSO_4_, and 0.25 mg/ml BSA were used. Reactions were run on the LightCycler 480 (Roche) with the following cycling conditions: 50 °C for 15 min (1 cycle); 95 °C for 5 min (1 cycle); 95 °C for 1 s and 60 °C for 20 s (45 cycles); and 40 °C for 30 s (1 cycle). A single fluorescence read was taken at the end of each 60 °C step, and a sample was considered positive if the Cq value was less than 40 cycles.

### Digital droplet PCR

For ddPCR analysis One-Step ddPCR Advanced Kit (Biorad, Hercules, CA) was utilized. 22 μl experiments were performed with 5 μl of extracted RNA, 5.5 μl of One-Step ddPCR supermix, and 2.2 μl polymerase mix. The assay specific primers and probes were used (listed in Table 1). Final concentrations of 0.7 μM primer, 0.25 μM probe, and 1 mM DTT were used. Droplets were generated on the QX200 AutoDG System (BioRad). Reaction droplets were PCR amplified under the following cycle conditions: 50 °C for 1 h (1 cycle); 95 °C for 10 min (1 cycle); 95 °C for 30 s and 60 °C for 1 min (40 cycles); and 95 °C for 10 min (1 cycle). Droplets were then run on QX200 Droplet Digital PCR system and analyzed using QuantaSoft v 1.7.4 software.

### Exclusivity/inclusivity panel

PTV and PICV probes and primers were tested against a panel of extracted viral nucleic acid samples to test assay specificity. The Unified Culture Collection (UCC) maintained at USAMRIID provided West Nile virus strain NY99 (UCC# Flavi022) and dengue virus serotypes 1–4 (UCC# Flavi029 strain WP74, UCC# Flavi030 strain 16803, UCC# Flavi031 strain CH53489, and UCC# Flavi032 strain 341750. respectively). Rift Valley fever virus, Lassa fever virus (strains Josiah, Weller, and Pinneo), Mozambique virus, Junín virus strain Espindola, Machupo virus strain Carvalo, Mobala virus strain Acar, and Heartland virus are all maintained at USAMRIID.

### Sanger sequencing

Capture regions were confirmed with Sanger sequencing by amplifying the capture region with modified M13F (-20) and M13R (-27) sequencing primers. Forward and reverse primers used for amplifying sequenceable PICV are as follows 5’-GTAAAACGACGGCCAGTGAATTGTAATACGACTCACTATAGGGCGAATTGAATTTAGCGGCCGCCATGTGTGGCCCCCATTT and 5’-CCTTTGTCGATACTGGTACATTACGCCAAGCTCAGAATTAACCCTCACTAAAGGGACTAGTCCTGCTCAGTTGTTAGGCAAAGTGGTCTT. Forward and reverse primers used for amplifying sequenceable PICV are as follows 5’-GTAAAACGACGGCCAGTGAATTGTAATACGACTCACTATAGGGCGAATTGAATTTAGCGGCCGCCAGATAGCTGCTGCCATTTTACA and 5’-CCTTTGTCGATACTGGTACATTACGCCAAGCTCAGAATTAACCCTCACTAAAGGGACTAGTCCTGCGCTTTTAAGTTTCCCAGCCAAA. Cycle sequencing was run with the BigDye® Terminator v3.1 Cycle Sequencing Kit (ThermoFisher), cleaned up with the Qiagen DyeEx 2.0 Spin kit (Qiagen), and run on the Applied Biosystems 3500xL Genetic Analyzer per manufacturers protocol.

### Statistical analysis

Cq values were calculated using the second derivative method with the Roche LightCycler 480 software version 1.5.1. GraphPad Prism v. 6.04 graphing software (GraphPad, La Jolla, CA) was used to plot sample data and for statistical analysis.

## Results and discussion

### Assay design/optimization

Initial evaluations identified optimal primer and probe concentrations for each assay combination, and preliminary downselection testing identified a final assay for each virus (Table 1). Assays were designed to target the polymerase genes due to their essential nature and stability, potentially limiting assay target erosion over time and avoiding potential heterogenetic differences between strains. Empirical testing determined an optimal annealing temperature of 60 °C with optimal primer and probe concentrations of 0.5 and 0.2 μM, respectively. Sanger sequencing was used to confirm the proper sequences were amplified during the real-time RT-qPCR reaction.

### Analytical limit of detection (LOD) determination

**Fig. 1:**
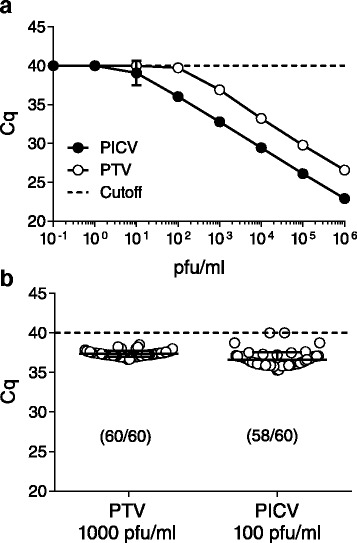
Analytical LOD for the PTV and PICV assays. Preliminary and confirmatory LODs were performed with primer probe combinations listed in Table 1. **a** Preliminary LODs were determined as having 3/3 replicate Cq values below 40 using serial 10-fold dilutions of PTV and PICV RNA extracted from water. Error bars represent the standard deviation of three replicates. **b** Confirmatory LODs were demonstrated with 60 replicates of PTV and PICV RNA extracted from water. Numbers in parenthesis represent the number of replicates with Cq values below the cutoff line. Each replicate is shown as an individual point with the bars representing the mean and standard deviation. In each assay, replicates that had no amplification curves or Cq values falling above 40 were given a base value of 40

To have confidence in assay sensitivity, we conducted analytical evaluations including a preliminary LOD and a confirmation of LOD using nucleic acid extracted from virus-infected Vero E6 cell culture supernatant using plaque forming units (PFU) as the unit of measurement. Testing of eight, 10-fold serial dilutions from each virus ranging from 10^6^ to 10^-1^ PFU/ml with each assay identified the preliminary LOD in which all three replicates were positive (Fig. 1a). This testing showed the preliminary LODs were 1000 PFU/ml and 100 PFU/ml for the PTV and PICV assay, respectively. In each case, we confirmed the preliminary LOD in a statistically robust manner (Fig. 1b) using 60 replicates at the preliminary LOD. For PTV, all 60 replicates fell below the cutoff with an average Cq of 37.73, and 58/60 replicates were positive for the PICV assay with an average Cq of 36.16. The coefficient of variation for PTV and PICV was 1.15 and 2.13 %, respectively. Exclusivity testing of primers and probes against a panel of extracted viral RNA listed in the methods were all negative. This panel included other Old and New World arenaviruses as well as phleboviruses in order to show differentiation between related viruses.

### Mock Clinical LOD determination

**Fig. 2:**
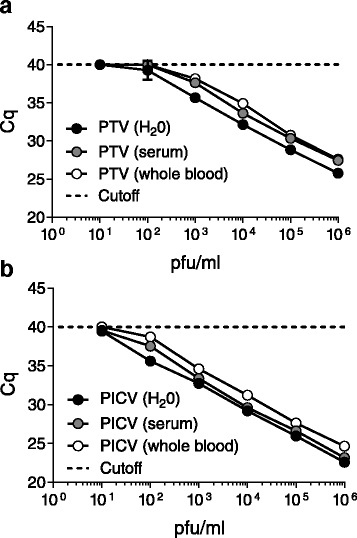
Mock clinical LODs for the PTV and PICV assays. Total RNA extracted from 10-fold serial dilutions of (**a**) PTV and (**b**) PICV spiked into water, sera, and whole blood were extracted in triplicate and tested with the final PTV and PICV assay listed in Table 1. In each assay, replicates that had no amplification curves or Cq values falling above 40 were given a base value of 40. Error bars represent the standard deviation of three replicates

Inhibitors of PCR are found in complex matrices such as blood [2] or stool [23, 28], and these inhibitors can carry through RNA preparation methods, affecting real-time RT-qPCR results and impacting assay sensitivity [16]. To further characterize these assays, we spiked each virus into water, human sera, and whole blood and performed six, 10-fold serial dilutions with matrix followed by nucleic acid extraction to re-establish preliminary LODs. Final pre-extraction concentrations ranged from 10^6^ to 10^1^ PFU/ml. For each matrix tested, water, serum, or whole blood, the LOD for PTV remained consistent at 1000 PFU/ml as determined by 3/3 replicates falling below the 40 Cq cutoff value (Fig. 2a). Similarly for PICV, the LOD remained unchanged when compared to the analytical characterizations at 100 PFU/ml for all three matrices tested (Fig. 2b). In both instances, evaluation criteria dictated Cq values greater than 40 be considered negative and given a final value of 40. Overall, these evaluations showed little to no impact or inhibition due to clinical matrix carryover for these assays.

The variation between PFU and RNA copy can vary widely depending on strain and growth conditions. Similarly PFU/ml is based on plaque assay and does not account for variations in the extraction efficiencies of sample preparation methods. To account for these variations, digital droplet PCR (ddPCR) was used to quantitatively determine post-extraction the number of target RNA molecules (counts) per μl of sample. To directly correlate ddPCR results to previous experiments, the spiked 10-fold dilutions of water from Fig. 2 were retested with ddPCR reaction chemistries (Additional file 1). These results demonstrate an LOD of 1000 PFU/ml for PTV and 100 PFU/ml for PICV correlate to 2 target counts/μl for PTV and 0.3 target counts/μl for PICV. Since 5 μl of sample is used per reaction, these data show detection limits as low as one RNA molecule per reaction.

## Conclusion

Both RVFV and LASV are highly pathogenic viruses of significant public health and economic concern, and both are considered biothreat agents. Use of RVFV is limited to BSL-3 conditions, and LASV is restricted to the highest level of containment, BSL-4. Due to these restrictions and the limited access to these viruses, BSL-2 models for both viruses have been developed for pathogenesis and therapeutic studies. To date, the authors are unaware of published real-time RT-qPCR assays for either PTV or PICV, so we developed and characterized real-time RT-qPCR assays for both PICV and PTV.

A series of primers and probes for each virus targeting the polymerase gene were optimized and tested for assay LOD and performance in multiple clinical matrices. Real time RT-qPCR analysis and ddPCR both demonstrate clinically relevant LODs, detecting as low as one RNA target molecule per reaction. These values are within the range of other real-time RT-qPCR assays developed by our group targeting filoviruses, arenaviruses, and New World hantaviruses [24]. These assays will help track viral kinetics when utilizing PTV and PICV as infection models in animal models. Since PTV can also clinically infect humans, this assay can be used for biosurveillance studies or for diagnostics.

## Additional file

Additional file 1: Absolute quantification of PTV and PICV RNA molecules using dd PCR. Viral cell culture supernatants spiked in water at specific PFU/ml were 10-fold serially diluted, extracted, and tested using the QX200 digital droplet system. Log_10_ transformation of PFU/ml of the spiked dilutions versus counts/μl determined by ddPCR are graphed. Linear regression analysis along with slope, R square value, and equation are shown. (PDF 112 kb)

## Abbreviations

BSL: Biosafety level
ddPCR: Digital droplet PCR
LASV: Lassa virus
LOD: Limit of detection
MGB: Minor groove binding
PICV: Pichinde virus
PTV: Punta Toro virus
RVFV: Rift Valley fever virus
