# Supplementary material for: Development of real-time reverse transcriptase qPCR assays for the detection of Punta Toro virus and Pichinde virus
Source: Virol J. 2016 Mar 31;13:54. doi: 10.1186/s12985-016-0509-3 (PMC4815133; doi:10.1186/s12985-016-0509-3)
Supplement: Additional file 1: — Absolute quantification of PTV and PICV RNA molecules using dd PCR. Viral cell culture supernatants spiked in water at specific PFU/ml were 10-fold serially diluted, extracted, and tested using the QX200 digital droplet system. Log10 transformation of PFU/ml of the spiked dilutions versus counts/μl determined by ddPCR are graphed. Linear regression analysis along with slope, R square value, and equation are shown. (PDF 112 kb) [file 12985_2016_509_MOESM1_ESM.pdf]

Additional file 1. Absolute quantification of PTV and PICV RNA molecules using dd PCR
Viral cell culture supernatant spiked in water at specific PFU/mls were 10-fold serially diluted, extracted, and tested using the QX200 digital droplet system. Log_{10} transformation of PFU/ml of the spiked dilutions versus counts/μl determined by ddPCR are graphed. Linear regression analysis along with slope, R square value, and equation are shown.

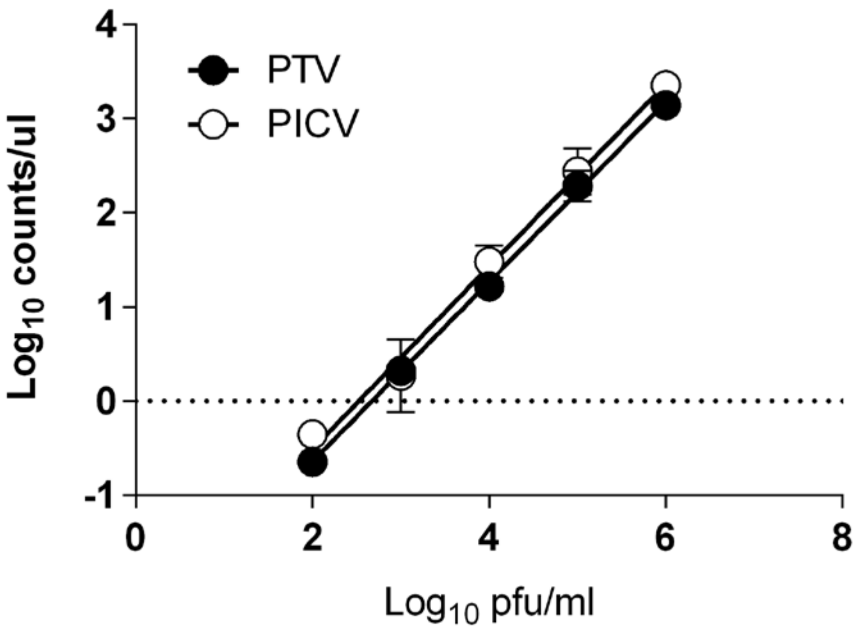

	PTV	PIVC
Slope	0.9542 ± 0.01945	0.9689 ± 0.04708
R square	0.9946	0.9725
Equation	Y = 0.9542*X - 2.551	Y = 0.9689*X - 2.447
